# Changing attitudes towards HIV testing and treatment among three generations of men in Cameroon: a qualitative analysis using the Fogg Behavior Model

**DOI:** 10.1186/s12889-023-15139-3

**Published:** 2023-03-15

**Authors:** Leila Katirayi, Patrice Tchendjou, Boris Tchounga, Muhamed Mbunka, Madison Wicks, Donaldson F. Conserve

**Affiliations:** 1 Research Department, Elizabeth Glaser Pediatric AIDS Foundation, 1350 Eye St NW, Suite 400, Washington, DC 20005 USA; 2 Programs Department, Elizabeth Glaser Pediatric AIDS Foundation, Yaoundé, Cameroon; 3 Research Department, Elizabeth Glaser Pediatric AIDS Foundation, Yaoundé, Cameroon; 4 Department of Anthropology, George Washington University, Washington, DC USA; 5 Milken Institute School of Public Health, George Washington University, Washington, DC USA

**Keywords:** Antiretroviral treatment, Cameroon, HIV, Masculinity, Men living with HIV, Social norms

## Abstract

**Introduction:**

Men are less likely than women to test for HIV and promptly initiate antiretroviral treatment, resulting in advanced HIV disease and increased mortality rates among them.

**Methods:**

In-depth interviews were conducted with men and leaders in the west and central regions of Cameroon. Men were recruited from existing community groups and stratified by age: 21–30 years, 31–40 years, and 41 years and older. Community leaders were recommended by the community dialogue structure chairman. Interviews were conducted using a semi-structured guide in English or French, depending on the participant’s preference. Transcripts were coded in the MAXQDA v.12 software and analyzed using thematic analysis and by age group. The Fogg Behavior Model was used to gain a deeper understanding of the different perceptions across all age groups.

**Results:**

Younger men (21–30 years) were generally more accepting of HIV testing, as it had become normative behavior. Although financial barriers could limit access, free testing was mentioned as a prompt to initiate HIV testing. The middle age men (31–40 years) had the most concerns about HIV testing interrupting their work day and recommended increasing testing locations and hours. The older men (41 + years) were the least motivated to get tested, citing worries about the impact on their social standing within the community. All age groups reported being motivated to begin treatment if they were found to be HIV-positive.

Participants also provided insights regarding community HIV testing and treatment messaging. Younger and older men preferred to hear directly from qualified health professionals, but younger men noted that social media, radio, and TV could be utilized. Middle age men also identified TV and radio as effective mediums, if door-to-door messaging was not an option.

**Conclusions:**

The study highlights important considerations when planning future information-sharing activities for HIV testing and treatment. Since lived experiences differ across generations and societal roles continue to change, not only should the content of messages differ among the generations, but the means of communication must also be considered to ensure the messages are conveyed through a trusted source.

## Introduction

UNAIDS estimates that men in sub-Saharan Africa are 20% less likely than women to know their HIV status and 27% less likely to begin treatment (On [1]. There are significant differences between HIV testing practices among men and women in Cameroon. Within Cameroon, 62% of men compared to 81% of women are aware of their status and only 43% of men living with HIV are on treatment, as opposed to 56% of HIV-positive women [2]. Data show men in Cameroon test for HIV less frequently than women across the age ranges. Approximately 66% of older men (40–44 years old) reported ever tested for HIV as compared to 72% of women. Additionally, 46% of younger adults (20-24 years old) reported ever receiving HIV testing compared to 73% of their female counterparts [3]. Approximately 90% of HIV infection in Cameroon is through heterosexual sex [4].

The gender disparity in HIV testing and treatment uptake has serious implications for the health outcomes of men. The delay in treatment initiation and high rates of treatment interruption has created a reduction in life expectancy of up to ten years for HIV-positive men and women [5].

Men are less likely than women to promptly test for HIV due to a combination of logistical, societal, and cultural beliefs. Men report challenges with wait times and issues with facility hours conflicting with their work hours [6]. Privacy and confidentiality are also well-reported concerns, with some men wanting to be tested away from their community [6]. Transportation is an additional well-documented barrier [7, 8].

Previous literature has documented that men feel the health facility is a female space, which discourages their utilization of it [9]. Men report not feeling comfortable or welcome at the health facility and that they would prefer to receive services elsewhere, such as at home [7]. One study suggested that a man-to-man approach to self-testing may be acceptable among men [10].

Testing HIV-positive and admitting illness (and essentially weakness) threatens traditional norms of masculinity (Camlin et al. [9]). Stigma is a well-documented barrier for both men and women, although it is experienced differently across the genders. A scoping review discussed how stigma affects the family-provider role, affecting their ability to provide for their family, achieve respect in their community, and attain a higher social status [11]. Illness and dependence on others threaten traditional male identities as the breadwinners and leaders of the family. The association of male strength with risk-taking behaviors and invulnerability can make men less likely to seek medical help when experiencing symptoms [12].

HIV messages have been shared throughout sub-Saharan Africa since the 1990s. As a result of the earlier HIV epidemic, before treatment was readily available and when HIV-related death was common, HIV became labeled a ‘death sentence’ [13]. Thirty years later, with the availability of treatment and fewer HIV-related deaths, the perceptions around HIV have shifted. To comprehend how men perceive HIV testing and treatment, it is critical to explore their thoughts among different age groups.

Minimal research has been conducted in Cameroon regarding men’s perspectives toward HIV testing. This study sought to gain a deeper understanding of the challenges men experience accepting HIV testing and treatment across three different age groups of men in Cameroon. The study also aimed to identify solutions to strengthen men’s acceptance and engagement in HIV testing and treatment by recognizing the motivations of each age group.

## Methods

### Study design

This study used an exploratory qualitative design. In-depth interviews (IDIs) were conducted with men and male community leaders in the selected regions of west and central Cameroon.

### Study population and recruitment

To ensure the study captured the different experiences and perceptions of men across various ages, men were stratified into three groups by age: 21–30 years, the ‘young’ men; 31–40, the ‘middle age’ men; and 41 years and older, the ‘older’ men. The minimum age of 21 years was selected because it is the age of the majority in Cameroon. The sample size included 18 IDIs with the young men, 14 IDIs with the middle age men, and 16 IDIs with the older men. Sampling was split equally across the two regions. Previous literature has indicated that saturation may be reached at 10–12 interviews [14].

Men were eligible to participate if their primary residence had been within the selected region for at least one year and if they had been sexually active with at least one partner. Men were recruited from existing men’s groups within the selected districts. The community chairman or the health area leader identified potential men’s groups in the community from which the desired age groups could be recruited. The study was presented to the men’s groups during their regular meetings by research assistants (RAs) and men interested were invited to volunteer for the study on a first-come, first-served basis. The RAs were onsite to enroll men immediately following the introduction of the study.

Male community leaders were eligible to participate if they had been a leader in the community for at least one year. Potential participants were identified and recommended by the community dialogue structure chairman or health area leader and were then subsequently introduced to the RAs. A total of five male community leaders were selected to participate from each region of study.

### Study sites

We selected two regions (the central and west regions) based on HIV prevalence, settings, and cultural diversity (Table 1). In each region, we selected the most densely populated district. In the central region, the district of Djoungolo was selected while the district of Mifi was selected in the west.

**Table 1:** Selection criteria for study sites

Criteria	Centre	West
Prevalence	High prevalence (5.8%)	Low prevalence (2.7%) [3]
Setting	Urban setting	Semi-urban and rural settings
Cutural diversity	Culturally represent three cultural zones in Cameroon (centre, east and South regions)	Cuturally represent three cultural zones in Cameroon (west, littoral, northwest, and southwest)

### Data collection

The IDIs used a semi-structured interview guide containing questions related to normative, behavioral, and cultural beliefs regarding men’s access to and use of HIV testing and treatment resources. Men were asked about their individual experiences, whereas the community leaders were asked to speak on behalf of the broader perceptions of the community. The IDIs were conducted by four male RAs, who had been trained by EGPAF staff, and were held within the community at a convenient time and location for the participant. Each IDI took place at a private location to ensure confidentiality, lasted approximately 45 min, and was conducted in English or French, whichever language the participant preferred. All IDIs were audio-recorded, transcribed, and translated from French to English by the RAs. Written informed consent was obtained before any data were collected. Data were collected August-October 2018.

### Analysis

Transcripts were reviewed by the study team to create a code list, which was then modified and updated throughout the coding process. The transcripts were coded by two RAs in the qualitative analysis software program MAXQDA v.12. Data reduction and summary table matrices were generated to help identify themes and key findings. Data were analyzed by age group and compared for generational differences. Data were also evaluated to assess the responses of the men against the community leaders.

### Theoretical application

To gain a deeper understanding of the different perceptions and motivations across the three age groups, data were analyzed using the Fogg Behavior Model [15]. This model examines the interaction of motivations, abilities, and prompts in changing human behavior. This model not only enlightens possible explanations for the observed behavior, but also provides insight into changing HIV testing and treatment behaviors over time.

**Fig. 1:**
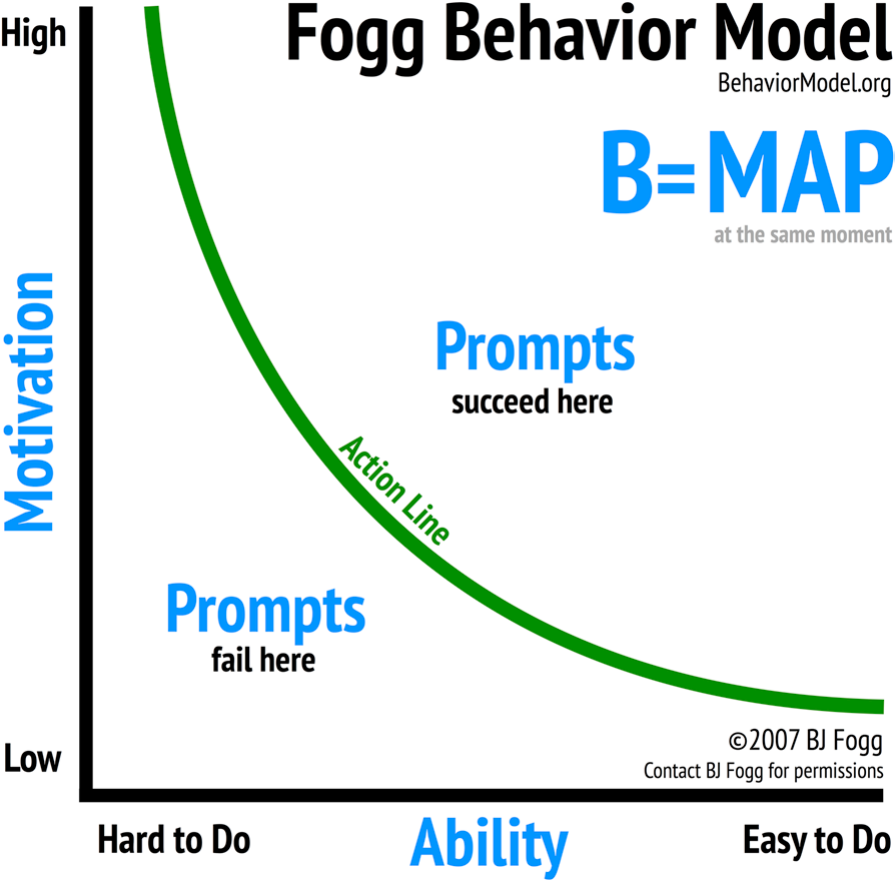
Fogg Behavior Model [15]

According to the Fogg model, motivations describe the desirability of actions and are qualified on a spectrum of low to high. They include distinctions between physical (pain/pleasure), emotional (hope/fear), and social (acceptance/rejection) consequences of the action. Abilities refer to the capability of an individual to perform a behavior and are labeled between easy and hard. Different aspects of ability include time, money, physical effort, mental effort, perceived adherence to social norms, and how well a behavior fits within an established personal routine. Lastly, prompts p are signals to an individual that tell them when it is the correct time to act and can push an individual's motivation and ability coordinates above the action line (see Fig. 1). Different prompts are utilized depending on the individual's relative motivation and ability.

Using the Fogg model, motivations and abilities can be represented graphically. An asymptotic action line delineates whether a behavior is performed, with above the line indicating it will be performed and below the line meaning it will not be performed (Fig. 1).

In this analysis, the salient factors affecting the motivations and abilities of Cameroonian men in the west and central regions are identified and explored.

## Results

A total of 48 IDIs were conducted with men and community leaders in the west and central regions, representing a variety of educational, marital, and professional statuses, in addition to ages (Tables 2 and 3). Information was also gathered on the participants’ prior testing experiences.

**Table 2:** Men's demographics by age group

	**Younger Men *N* = 18 n (%)**	**Middle age men *N* = 14 n (%)**	**Older men *N* = 16 n (%)**
**Mean age, years**	26.5	35.8	55.3
**Highest education level**			
Primary school	0	5 (36)	2 (12.5)
Secondary school	11 (61)	4 (28)	11 (68.75)
University	7 (39)	5 (36)	3 (18.75)
**Marital status**			
Never married	14 (78)	5 (36)	0 (0)
Live-in partner (unmarried)	3 (17)	2 (14)	0 (0)
Married (first wife)	0 (0)	5 (36)	11 (68.75)
Married (polygamous)	1 (6)	1 (7)	4 (25)
Separated	0 (0)	1 (7)	1 (6.25)
**Employment rate**	50%	78.6%	87.5%
**Testing participation (self-reported)**	15 (83.3)	14 (100)	13 (81.25)
Mobile testing	4 (22.2)	3 (21.4)	3 (18.75)
Facility testing	11 (61.1)	11 (78.6)	10 (62.5)
Not tested	3 (16.7)	0 (0)	3 (18.75)

**Table 3:** Community leaders’ demographics

	**Community leaders *N* = 10 n (%)**
**Mean age, years**	54.3
**Highest education level**	
Primary school	1 (10)
Secondary school	2 (20)
University	5 (50)
Unknown	2 (20)
**Average years in leadership**	13.7
**Leadership position**	
Traditional	4 (40)
Association	4 (40)
Religious/Spiritual	2 (20)

### Motivations

Motivation levels of the various age groups were largely determined by the individuals’ understanding of HIV’s prognosis and the potential social consequences of testing. The younger men generally believed that because HIV could be managed and did not drastically limit one’s life, early detection was a source of hope and motivation for testing. Additionally, this age group perceived testing as not only socially acceptable, but also responsible and necessary for the safety of their communities. Most of these participants expressed that knowing one’s status is a component of responsible sexual behavior and noted they had an obligation to protect their partners.

The middle age men exhibited a range of motivations particular to their life circumstances. Social acceptance and rejection were competing influences on this age group’s motivations. Several of the men expressed testing was becoming increasingly necessary to secure a marriage. However, a positive result could greatly impede a man’s marriage potential or erode his standing in his family and community. These competing social consequences of testing produced a moderate motivation level for testing for this age group.

The older men were the least motivated to pursue testing. While some perceived HIV to be a death sentence with severe health consequences, virtually all of the participants in this age group believed a positive result would threaten their standing and respect in their communities. Overall, this group was unmotivated to seek testing and preferred to stay ignorant of their HIV status rather than face the potential social consequences. This mentality was echoed by community leaders who identified social ramifications as one of the primary testing deterrents for men in general.

All age groups reported being motivated to begin antiretroviral treatment (ART) if they were found to be HIV-positive. Participants expressed great optimism about the success of ART and its ability to ensure a relatively normal life with limited health concerns. Older participants expressed more apprehension around the stigmatization of treatment but still said they would initiate ART if they tested HIV-positive. Table 4 provides a summary of each age group’s motivations.

**Table 4:** Men’s motivations by age group

Age group	Observed theme	Quote
Young men	**HIGH motivation level** Hope: HIV was seen as treatable and it was possible to lead a full and happy life with HIV Social acceptance: testing and treatment were responsible actions	“(When taking ART) It's like you did not even contract HIV. You continue to live a normal life.” (Young man, centre, ID: C-M-76–07)
Middle age men	**MODERATE motivation level** Social acceptance/rejection: testing and treatment may protect a man’s family from physical harm, but a positive status could threaten marriage prospects	“If you do not want to do the test, they will cancel the marriage. The marriage will be canceled. It is unavoidable.” (Middle age man, west, ID: W-M-93–11)
Older men	**LOW motivation level** Fear: a positive HIV status was viewed as a death sentence Social rejection: a positive test could threaten a man’s standing in his family and community	“They feel ashamed because if the test shows positive, his surrounding will see him as someone who is not prudent, someone who is promiscuous. The fear is that the test may show positive, the shame is that when it is positive what will the people around him think of him.” (Older man, centre, ID:C-M-76–30)

### Abilities

Perceived ability levels regarding testing were largely influenced by the economic security of each age group. Virtually all young men experienced or perceived significant financial impediments to their ability to access testing and treatment. Young men reported that the price of medical services, as well as associated costs such as transportation, could be a deterrent from seeking HIV testing. Young men also had limited free time to go to clinics or testing campaigns, which often occurred during work hours. Although most community leaders shared that there were resources in place to address such barriers, many also recognized that testing fees, limited clinic hours, and associated costs like transportation did prevent some people in the community from accessing testing.

Middle age men reported having better access to financial resources that made testing affordable compared to the men in the 21–30 year age group. However, the middle age men still faced barriers in finding time to visit the health clinics.

Older men viewed HIV health services as affordable and also espoused the value that responsible men must always make time for their health if they might be sick.

Although issues with treatment costs and time barriers were reported, the vast majority of participants across all age groups said they would start ART right away, regardless of these challenges. Table 5 provides a summary of each age group’s abilities.

**Table 5:** Men's abilities by age group

Age group	Observed theme	Quote
Young men	**LOW ability** Time: testing and treatment required medical visits, which interfered with work hours Money: testing and treatment were perceived as having high monetary costs	“The difficulty would be at the level of paying for the test. They may tell you that maybe the test is 100,000 XAF [160 USD], and your monthly salary is 50,000 XAF [80 USD]. So you would have to work for more than two months to have the money for the test. Because if you have a wife you need to feed her and pay bills. So it is a saving that you have to do for about a year.” (Young man, centre, ID: C-M-75–37)
Middle age men	**MODERATE ability** Time: testing and treatment required medical visits, which interfered with work hours Money: there were resources available to cover the cost of testing and treatment	“Going to the hospital and stand on a long endless line to do the test is not easy. It is not something you can do for 30 min…you would have to go and [wait] for them to start testing, you most have to spend a lot of time there.” (Middle age man, centre, ID: C-M-76–39)
Older men	**HIGH ability** Time: time could and should be made to address health concerns Money: testing and treatment was affordable	“Some find this price quite high. For me, it is not a problem. And even when I pay these fees will be 100% reimbursed by my insurance. So, there is no reason I cannot get tested for HIV.” (Older man, centre, ID: C-M-75–03)

## Prompts

When asked what would make testing more accessible, young men identified facilitator prompts that would increase their ability to get tested. The most common recommendation, provided by most of the young men, was to make free testing readily available. They also suggested increasing the number of local clinics that men could access.

Middle age men also identified prompts that would increase their access to HIV testing and treatment services. This age group primarily focused their testing recommendations on increasing the number of clinics and extending their hours, as well as positioning the testing centers in more convenient locations for working men. Additionally, many participants advocated for optimistic messaging that would motivate men by destigmatizing testing and challenging misconceptions surrounding HIV-positive prognoses. Several middle age men suggested that increased confidentiality protections would also make men less fearful of testing. Similarly, the community leaders recommended improving the current functioning of clinics to better protect men’s privacy.

The older men emphasized prompts that would decrease the stigma surrounding testing for HIV, helping them avoid potentially threatening their social status. The vast majority of these men recommended normalizing discussions of HIV testing and treatment through education and messaging, particularly by emphasizing the health benefits of early detection and how testing can protect one’s family.

Participants also provided insights regarding how messages were best spread to the community. Most younger men expressed a preference for hearing information directly from qualified health professionals but also noted social media, radio, and TV could be utilized, although radio and TV tended to be associated with older generations by this younger group. Middle age men also identified TV and radio as effective mediums if door-to-door messaging was not an option. Similar to the young men, the older men identified health workers as the best conveyors of information to communities. Community leaders were the only group to primarily offer themselves at their group meetings as the primary source of HIV messaging and many of them expressed doubts regarding the use of media (i.e. TV, radio, or social media). Table 6 provides a summary of each age group’s prompts.

**Table 6:** Men’s prompts by age group

Age group	Prompts	Quote
Younger men	Increase the accessibility of testing and make testing free	“ok for me, they should do it for free. If they do it for free, many would come, but only those who are interested. For us in Africa, ok in Cameroon, we like anything that is free, it would encourage many people to go and get tested… I would do it during the weekend on Sunday.” (Young man, centre, ID: C-M-75–37)
Middle age men	Increase one’s ability and motivation to pursue testing and treatment	“So, you can create kiosks open seven days a week at all Carrefour [small gathering spot in the community usually at crossroad] for these tests to be permanent. There, I'm sure everyone will have the time. Everyone will become aware because everywhere he goes he will find a kiosk. In this way, we will defeat HIV here.” (Middle age man, centre, ID: C-M-75–11)
Older men	Increase one’s motivation to pursue testing and treatment	“If by chance …[they are infected] there is a way out…. To show them, to convince them, to prove to them that there are possibilities to get out and easily too. And that staying without seeking to know one’s status is not a good thing, it’s not a guarantee, not for them, not for their children. To tell them that it is important to know for the sake of the children. it is important to know one’s HIV status.” (Older man, west, ID: W-M-93–36)

## Discussion

The study revealed the different motivations, abilities, and prompts for seeking HIV testing and treatment among men in Cameroon. Overall, men were motivated to seek HIV testing for a variety of reasons, including the fact that if HIV was detected and treated early, it would be manageable, as well as the idea of needing to protect one’s partner and community. However, barriers (stigma and the perception HIV is a death sentence, as well as the cost of transportation) reduced men’s motivations and abilities to test for HIV. The prompts, or recommendations, the participants reported that would increase their motivations and abilities to test for HIV were to make free HIV testing more accessible, increase the number of testing clinics, and support more campaigns that focus on optimistic messaging to destigmatize testing and challenge misconceptions surrounding HIV. In addition, men identified several methods of how hopeful messages about HIV could be diffused throughout their community and networks, including social media, TV, door-to-door messaging, and health professionals. While these findings parallel those of other studies that investigated the facilitators and barriers of HIV services among men in sub-Saharan Africa  [10, 16–22]) our results provide a more nuanced qualitative perspective of how men’s motivations, abilities, and prompts for HIV services vary by age. These findings warrant the need for intervention strategies and messages to account for men’s age to ensure the programs are relevant for men of different age groups.

Within this study, younger men perceived HIV to be manageable and believed a person should be able to live a normal life if they were diagnosed with HIV early. This observation is supported by a study conducted in Cameroon, which found younger men, compared to women, were less likely to be worried about the negative consequences of HIV and AIDS [23]. This phenomenon may be explained by the intentional change in global and local campaign messages distributed through different media platforms about HIV in recent years that discourage discrimination and stigma and reinforced the importance of HIV testing, support, and acceptance of people living with HIV [24–26]. Younger men are more likely to be exposed to the positive messages about HIV and witness people living with HIV who are healthy and living normal lives [27, 28]), compared to older men who were exposed to stigmatized and outdated messages about HIV and ART during the earlier period of the HIV epidemic, when lifesaving treatments were not yet widely and freely available [29]. For example, the universal “Test and Treat” and “Undetectable = Untransmissible” HIV campaign strategies, which encouraged early testing, immediate treatment initiation, and viral suppression for people diagnosed with HIV, only began to be implemented in the past decade [30, 31]. This increased the likelihood for younger men to receive optimistic messages about HIV and treatment, compared to older men who may be holding on to outdated and more fearful messages.

Related to this phenomenon, younger men were the only group that expressed social media was a viable option for how to spread information about HIV in their community. This finding resonates with research that has shown high engagement of young men on social media for the submission of ideas on how to increase HIV testing among men in Eswatini [21]. Additional studies conducted in other regions have provided evidence for how social media can be used to disseminate information about HIV, including information on HIV testing and guidance on how to order HIV self-testing kits [32, 33], which allow people to test at home and has been shown to be preferred among men, including younger men [34–36].

Another difference based on men’s ages was the concern raised among the middle age men about the role of HIV testing before marriage and how a positive HIV test result could impede their chances of marriage. This concern stems from the pre-marital HIV testing conditions that some Pentecostal churches have instituted in Cameroon [37], which is similar to a few other African countries [38]. Pre-marital HIV testing has been adopted by several religious institutions and local governments as a strategy to increase HIV testing and prevent HIV transmission among couples [39–41]. Understandably, the middle age men would be more concerned about the impact HIV testing could have on their marriage potential, as men in this age group may be readier to settle down, compared to younger men who may not be financially ready for the responsibilities of marriage. However, Cameroonian men, regardless of age, were less likely than women to agree to marry a partner who was living with HIV if they tested HIV-negative [37]. Thus, the concern about a marriage proposal being rejected by a female partner if the male tested positive was similar to how many men in Cameroon would respond if they were negative and the woman was positive. This potential rejection fuels the fear of testing positive for HIV that the men in this age group reported. The fear of testing positive has been reported by men of different age groups in sub-Saharan Africa [12, 13, 42] and it has been the main objective of many HIV testing campaigns to address. Numerous campaigns focus on encouraging men to test and know their status and emphasize that a positive HIV status is not a death sentence and only requires HIV treatment, which can help a person to live a long and normal life [43–45]. In Cote d’Ivoire, a community-based behavior intervention for men called the “Brothers for Life” was adapted to address men’s fears about HIV testing and treatment and it transformed men’s HIV-related perceptions and behaviors, with 81% of men reporting to have tested and 100% of men diagnosed with HIV initiating treatment  [43].

In regards to the older men, they perceived HIV as a death sentence and were the least motivated to test for HIV due to the potential loss of respect in their communities if they were to test positive. These findings are consistent with other studies that have reported older and wealthy men’s concerns about losing their social status and community standing serve as barriers to HIV testing [16]. Older men are likely to be more established and enjoy the social and economic comfort they have achieved with age and may believe they have more to lose if seen testing for HIV and being diagnosed with HIV [16, 46]. In addition, older men’s view of HIV as a death sentence is supported by other studies and may stem from the old messages during the early days of the epidemic, before lifesaving ART was available  [16, 29, 46]. The need for reducing community stigma to promote HIV testing among men in this age group aligns with the higher rate of HIV testing found among men who did not perceive community-level HIV stigma [47, 48]. In contrast, higher community-level perceived stigma was found to be associated with higher testing frequency among men in Uganda, whereas lower individual-level perceived stigma was associated with higher testing [49]. Decreasing community-level stigma is crucial since studies have found that a majority of people living with HIV experience HIV stigma [50–52]. Despite the several studies reporting on the stigma associated with living with HIV in Cameroon, only one HIV stigma reduction intervention, “My Friend with HIV Remains a Friend”, was conducted in Cameroon, according to the literature, with more focus on secondary school students [53]. Findings of this study therefore suggested the need for more HIV stigma interventions at community-levels, including emphasis on communication and strategy to address the impact of old messages share in the early days of the epidemic. In the current context of availability of long-life ART treatment, communications should address the needs and perceptions of the three age categories of men which are definitely different.

### Limitations

The stratification of men across different age groups provided the study team with an in-depth understanding of the different perceptions and motivations across the generations. Conducting the study in the community, as opposed to in the health facility, may have facilitated men to feel more comfortable speaking freely. However, this study did not gather any data from the healthcare workers administering HIV tests or treatment, who may have provided additional valuable information to strengthen men’s acceptance of HIV testing and treatment. In addition, the selection of community leaders by the community chairman could have introduced a level of bias, however, this was the only cultural and social entry into the community.

## Conclusions

The study highlights important considerations when planning future public health communication/messaging for HIV testing and treatment. Lived experiences differ across generations and societal roles are changing. Messaging content must be designed to address the needs of these unique generations. Younger men want more focus on access to free testing and knowledge of free testing. Middle-aged men are more career-focused and want to improve access to HIV services by introducing more convenient clinic locations and hours. Older men are fearful of the social implications of an HIV-positive diagnosis so increased knowledge-sharing and stigma reduction efforts are needed. Not only should the content of messages differ among these populations, but the means of sharing information also need to align with generational differences to ensure men receive the messages through a trusted source. It is important to acknowledge variations across the generations and consider the opportunity this creates to most-effectively address each group’s unique needs.

## Data Availability

Data are available upon reasonable request to the corresponding author.

## Abbreviations

ART: Antiretroviral treatment
EGPAF: Elizabeth Glaser Pediatric AIDS Foundation
IDI: In-depth interview
IRB: Institutional review board
RA: Research assistant
